# Exploring the biophysical option space for feeding the world without deforestation

**DOI:** 10.1038/ncomms11382

**Published:** 2016-04-19

**Authors:** Karl-Heinz Erb, Christian Lauk, Thomas Kastner, Andreas Mayer, Michaela C. Theurl, Helmut Haberl

**Affiliations:** 1 Institute of Social Ecology, Vienna, Alpen-Adria Universitaet Klagenfurt, Vienna, Graz, Schottenfeldgasse 29, Vienna 1070, Austria; 2 Research Institute of Organic Agriculture, FiBL Austria, Doblhoffgasse 7/10, Vienna 1010, Austria

## Abstract

Safeguarding the world's remaining forests is a high-priority goal. We assess the biophysical option space for feeding the world in 2050 in a hypothetical zero-deforestation world. We systematically combine realistic assumptions on future yields, agricultural areas, livestock feed and human diets. For each scenario, we determine whether the supply of crop products meets the demand and whether the grazing intensity stays within plausible limits. We find that many options exist to meet the global food supply in 2050 without deforestation, even at low crop-yield levels. Within the option space, individual scenarios differ greatly in terms of biomass harvest, cropland demand and grazing intensity, depending primarily on the quantitative and qualitative aspects of human diets. Grazing constraints strongly limit the option space. Without the option to encroach into natural or semi-natural land, trade volumes will rise in scenarios with globally converging diets, thereby decreasing the food self-sufficiency of many developing regions.

## Abstract (web summary)

Safeguarding existing forests is an important ecological concern but constrains the expansion of farmland to feed the growing world population. Here the authors analyse the option space for future changes in agriculture and diets compatible with a no-deforestation goal.

## Graphical abstract

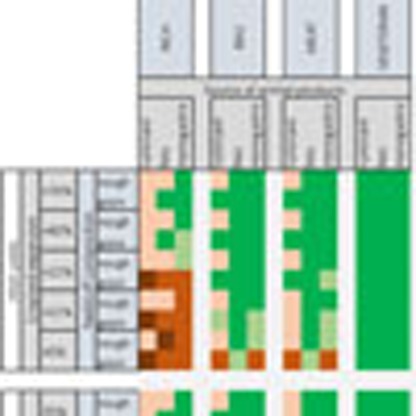

Future land use faces several interconnected challenges. Terrestrial ecosystems play a key role in the global climate system, host a substantial fraction of global biodiversity and provide ecosystems services that are essential for humans, including food, fibre, energy, water and air purification, micro-climate regulation and protection from natural hazards^1^. Three-quarters of the earth's terrestrial, ice-free surface is currently under human use^2^,^3^, and one-quarter of global potential net primary production (NPP_pot_, the annual net production of organic matter by primary producers such as plants that would prevail in the absence of humans) is appropriated by humans^4^,^5^. Land use is associated with many other environmental effects, such as eutrophication, pollution, biodiversity loss or climate effects, reaching levels that jeopardize the provision of ecosystem services to society^6^,^7^. Exploring ways that allow feeding and fuelling the growing global population while safeguarding the life-supporting functions of ecosystems is generally recognized as an urgent sustainability challenge^1^,^8^.

Protecting the remaining forested ecosystems is a central desideratum in this context. Forests store more carbon than any other land-cover type per unit area^9^ and host a considerable fraction of the global biodiversity^10^. A sizeable fraction of global pristine forests has already been converted into agricultural areas^2^, and this process is ongoing, particularly in tropical regions^11^,^12^. International activities such as the UN collaborative program ‘Reducing Emissions from Deforestation and Forest Degradation in Developing Countries' (REDD) aim at protecting the remaining forest ecosystems for climate-change mitigation targets. The global extent of forests is also proposed as an indicator for delineating a safe operating space for humanity, and its significance in the context of sustainability is highlighted^13^.

However, safeguarding the existing forests constrains agricultural development as it limits the expansion of cropland and grazing areas. Consequently, the increasing demand for food, feed, fibre and fuel of a growing world population^14^,^15^,^16^ will have to be met on shrinking per capita land areas. Recent studies suggest that providing sufficient food without cropland expansion is possible^17^, for example, by increasing yields above the current rates^18^, based on higher agricultural inputs and associated with massive trade volumes^19^. In contrast, other studies argue that low-input farming systems, such as organic agriculture, are vital for safeguarding important ecosystem services^20^,^21^,^22^. Whether and under which conditions low-input or organic agricultural systems are able to feed the world is fiercely debated^23^,^24^, warranting further investigation^25^. In the context of studies assessing the strategies of land-based climate-change mitigation, the future development of human diets^26^,^27^ and efficiency gains in global livestock systems^28^,^29^,^30^,^31^ have been identified as crucial. However, because of their large feed requirements, efficiency gains in livestock systems that are based on the enhanced use of cropland-derived concentrate feedstuff can also aggravate land competition for food production^8^,^28^,^32^. In contrast, the use of land that is not directly usable for food production with ruminants has an important potential to contribute to food security and the maintenance of livelihoods in specific contexts^33^,^34^,^35^. This plurality of viewpoints warrants a systematic exploration of the interplay of factors such as diets, yields and livestock efficiencies for agricultural development.

In particular, constraints to grazing become decisive in a zero-deforestation world. In that situation, cropland expansion can expand to unused, unforested land—which is typically of low productivity due to climate constraints and of high nature value (then, its conversion entails high ecological costs)—or, more likely, to other areas, that is, areas not used for cropping and infrastructure. Most of these areas are used for grazing, albeit at varying intensities^2^,^36^,^37^.

In this study, we systematically explore the options and constraints resulting from a hypothetical zero-deforestation boundary condition for agricultural production, thereby explicitly assessing limitations to grazing. We explore the individual role of supply-side measures (including cropland output intensification and cropland expansion), efficiency measures in the livestock system (increases in feed-conversion ratios) and demand-side measures (quantitative and qualitative changes in the human diet). For each of these parameters, we collected published forecasts for 2050 and incorporated them into a consistent biomass balance model (BioBaM; Supplementary Methods)^38^,^39^ to assess their combined effects. For each scenario which is a unique combination of individual variants of five parameters (Fig. 1), it calculates biomass demand and supply balances—for the globe and for 11 world regions—along with the average grazing intensity and regional biophysical trade balances.

We assessed the feasibility of 500 scenarios. ‘Feasibility' was defined as a situation in which global food demand is matched by cropland supply, and livestock grazing intensity stays within ecological thresholds. Trade is assumed to balance deficits of regional production and consumption for all feasible scenarios, assuming no trade barriers exist. The option space is defined as the sum of all feasible scenarios.

Our analysis reveals that a large range of options exist to feed a no-deforestation world. Nearly two-thirds of the 500 calculated scenarios are classified as ‘feasible' or ‘probably feasible,' even with low cropland-yield levels or RICH diets, but not when these two are combined. Cropland constraints and grazing constraints are approximately equally frequent. Biomass harvest, cropland demand and grazing intensity vary broadly within the option space, largely depending on diets. Grazing constraints strongly limit the option space in a world with moderate to high cropland expansion. Within the option space, trade volumes will rise if a more regionally equal per capita diet is adopted and no encroachment of farming into natural or semi-natural land is assumed.

## Results

### General results

**Figure 2:**
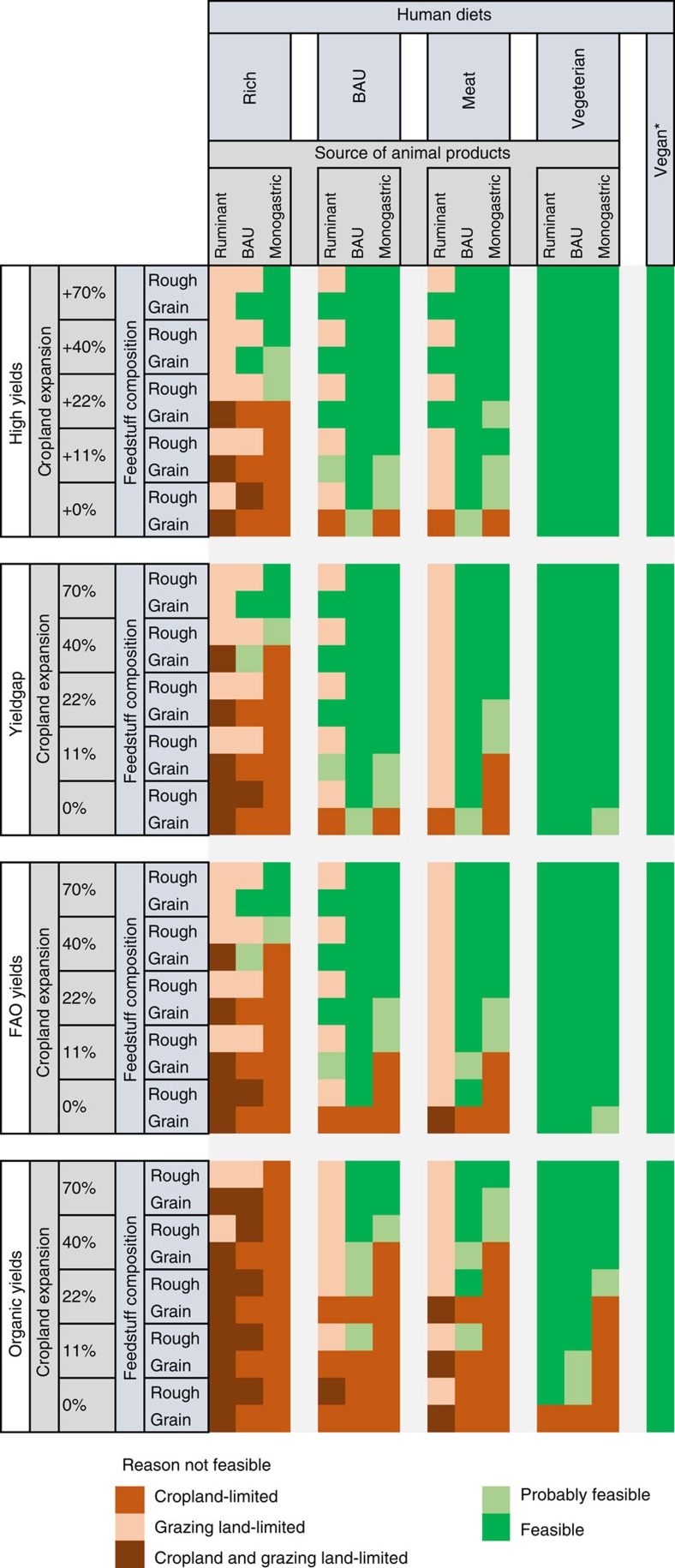
The biophysical option space in 2050. The option space (green cells) results from the combination of four yield variants, five cropland expansion variants, two variants of the feed basis for livestock at the supply side (lines) and five human diet variants combined with three variants of the origin of livestock products at the demand side (columns). Each cell in the option space represents a scenario. Scenarios affected by cropland limitations represent cases where the global demand for cropland products exceeds global supply by more than 5% and scenarios affected by grazing limitations when global grazing intensity exceeds ecological thresholds. ‘Probably feasible' are scenarios for which demand and supply differ by <5%. *For the VEGAN diet, the source of livestock products is not relevant.

An overview of the option space according to our scenario calculations is displayed in Fig. 2. More than 40% of all scenarios (211) are not feasible. Eighteen per cent of all scenarios are limited by cropland availability, 16% by limits to grazing intensity and 7% by a concomitance of both constraints. Whereas all VEGAN scenarios and 94% of the VEGETARIAN scenarios are feasible, approximately two-thirds of the BAU diet and only a small fraction of the RICH diet are found to be feasible (15%). With high yield levels, 71% of all scenarios are feasible (or probably feasible), compared with only 39% if organic yields are assumed. Apparently, the increased area demand resulting from low yields renders scenarios with richer diets unfeasible. The expansion of cropland into grazing areas enlarges the option space, but grazing constraints become increasingly important. The RICH diet combined with intermediate yield levels is feasible only in cases where a large share of high-quality grazing land is converted into cropland. Such scenarios, however, are often constrained by thresholds related to grazing intensities, owing to the decreased grazing land availability following cropland expansion.

### Factors determining the option space

**Figure 3:**
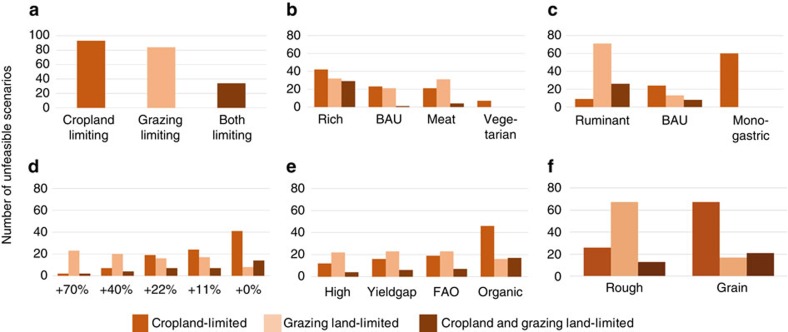
Grazing and cropland constraints. (**a**) Total number of unfeasible scenarios, broken down into (**b**) human diets (note that no constraints relate to the VEGAN variant), (**c**) mix of livestock products in human diets, (**d**) cropland expansion, (**e**) cropland yields and (**f**) composition of feedstuff. *Y* axis: number of unfeasible scenarios. For abbreviations, see the text and the caption of Fig. 1.

Figure 3 displays the relationship between cropland and grazing constraints and the individual parameters. This illustration highlights the strong effect of grazing constraints on the option space, particularly in response to cropland expansion.

Cropland constraints are closely connected to yield levels and cropland expansion variants. Low yields imply that a high number of scenarios are constrained by the availability of cropland, which hold true particularly for the ORGANIC yield variant. Grazing constraints depend on the origin of livestock products in human diets and the feedstuff composition. Apparently, the effect of reduced grazing area availability can be compensated for by lower demand for livestock products and by the higher reliance of livestock feeding on cropland-based products.

The results suggest that roughage-based livestock systems are primarily confronted with grazing constraints, whereas grain-based livestock feeding faces cropland constraints. Ruminant-based supply systems are primarily affected by grazing limits; monogastric pathways are more affected by cropland limits.

Human diets play a decisive role in the option space: Vegetarian or vegan diets are less often restricted by cropland availability or limits on the biomass supply of grazing land. Richer diets and cropland expansion are approximately evenly limiting. The two meat-based diets, BAU and MEAT, are similar with regard to cropland limits, despite the differences in global average calorie supply. However, they show distinct differences with regard to the limits posed by grazing. The MEAT diet, characterized by a larger demand for livestock products than the world average of the BAU diet variant (but a demand substantially lower than today's levels in industrial countries; Supplementary Tables 1 and 2), is strongly affected by grazing limits, although it has a smaller overall calorie supply.

In general terms, ruminant-based diets show a much smaller option space in the MEAT and BAU variants, constrained by limits to grazing. However, Fig. 2 reveals a noteworthy detail. Under conditions of low yields and small cropland areas, ruminant-based diets have an advantage over monogastric-based diets. In these cases, ruminants use resources that do not compete for cropland that is, in this scenario group, limited.

### Characterization of feasible and probably feasible scenarios

**Figure 4:**
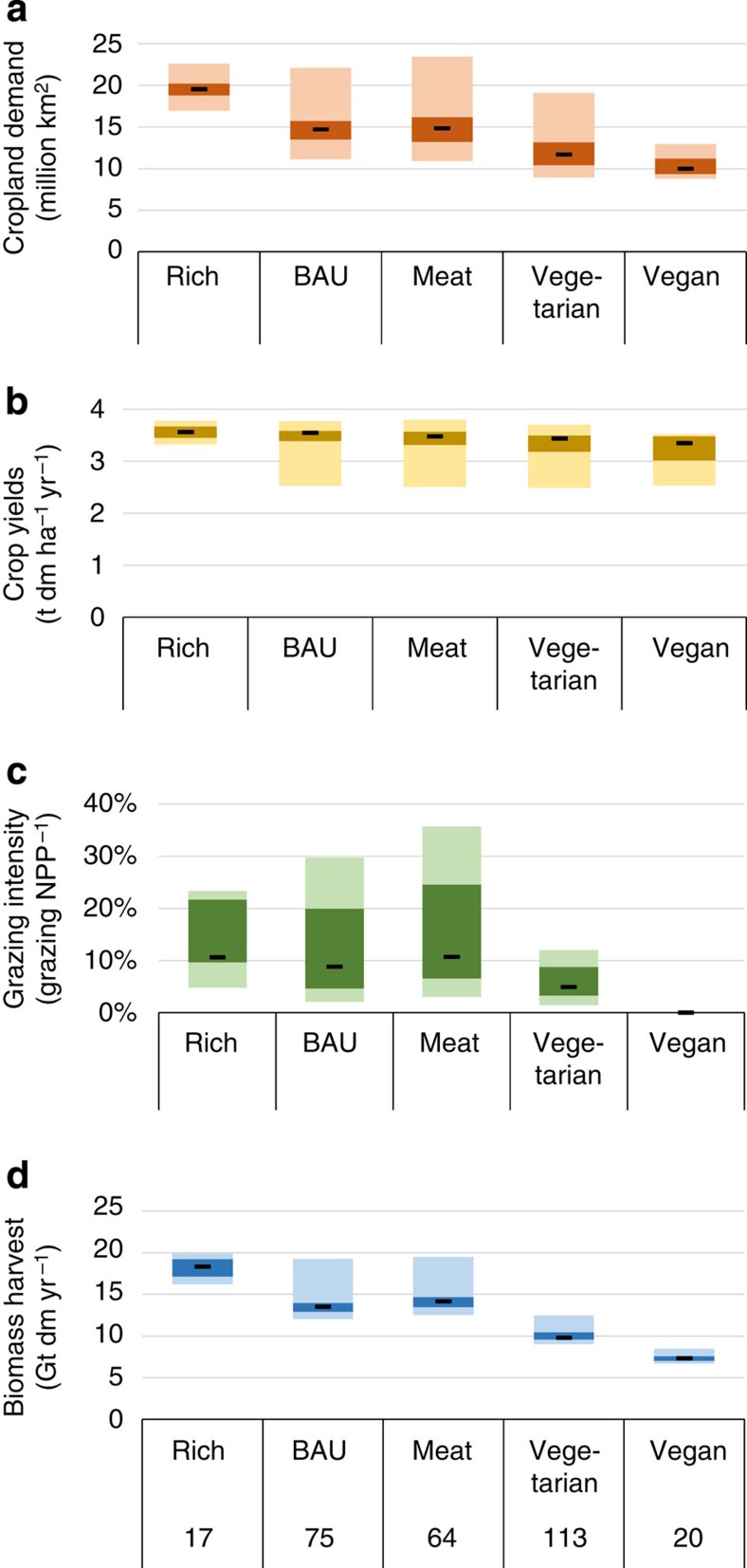
Characterization of feasible and probably feasible scenarios, broken down into human diets. (**a**) Cropland demand, (**b**) average cropland yields, (**c**) grazing intensity, measured as grazing harvest in per cent of actual NPP and (**d**) biomass harvest, that is, total biomass harvested globally for food supply, given as dry matter. Dark boxes indicate the two inner quartiles (>25 and <75%) of all feasible scenarios; light grey-shaded boxes indicate the minimum and maximum values. Small lines indicate the median. Numbers below diets indicate the number of feasible scenarios. The total number of scenarios per diet is 120, with the exception of the VEGAN diet, with 40 scenarios. For abbreviations, see the text and the caption of Fig. 1. Colour codes are aimed to facilitate comparison with Supplementary Figs 3–6.

The feasible and probably feasible scenarios vary strongly with regard to cropland demand, crop yields, grazing intensity and biomass harvest. The results for these parameters for all feasible scenarios are displayed in Fig. 4 in a breakdown into human diets (for other aggregations, see Supplementary Figs 3–6). Cropland demand shows a huge variation across all feasible scenarios (Fig. 4a). Whereas the VEGAN diets require less cropland than in the year 2000, the BAU and MEAT diets reach a cropland demand up to 23.5 Mkm^2^, 52% above the current levels. In contrast, the maximum cropland demand of the RICH diet is similar to the maximum of the BAU diets. However, here, the number of feasible scenarios is considerably reduced by grazing constraints. RICH diets are feasible only with considerable cropland expansion and high cropland yields (Fig. 4b), whereas the other diet variants are also feasible with low or moderate levels. Crop yields do not vary strongly between diet variants (factor 1.5 between lowest and highest; Fig. 4b).

Grazing intensity is highly variable in the option space and strongly dependent on diets, ranging from 0 to 36% of actual NPP (Fig. 4c). No grazing is associated with the purely plant-based human diet. Low values of grazing intensity are found in the VEGETARIAN diets, and the highest grazing intensities are found in the MEAT diets.

Unsurprisingly, biomass harvest varies by a factor of ∼3 among the diet variants. Note, however, that the variation of harvest within each diet group purely results from differences in livestock systems, that is, feed composition and the fraction of livestock products in human diets. The RICH diet variant is not feasible with ruminant products (Supplementary Fig. 7).

### Emerging trade patterns

**Figure 5:**
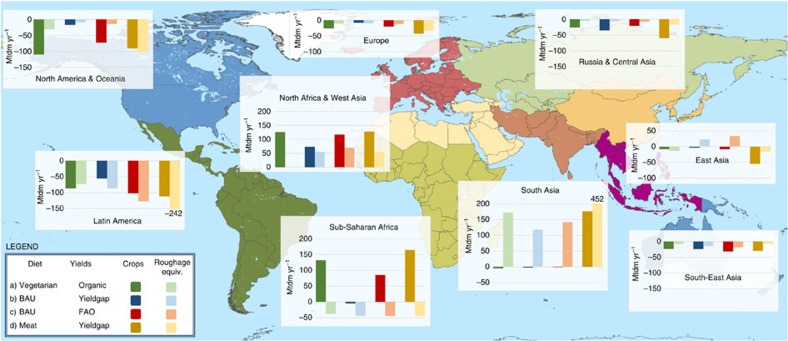
Regional net trade of selected scenario groups. Values refer to the median within a group. Positive values indicate net imports; negative values indicate net exports. Unit: million tons dry matter biomass year^−1^, primary biomass equivalents. Note the truncated *y* axis for South Asia and Latin America. Trade of livestock products is expressed as the equivalent of roughage that would be required to sustain domestic ruminant production.

The scenarios differ strongly in their regional self-sufficiency rates (that is, the ratio of domestic extraction and consumption). In feasible and probably feasible scenarios, regional deficits are balanced by imports from surplus regions. Figure 5 displays the resulting net trade patterns according to four scenario groups. Group (a) includes all feasible VEGETARIAN diet scenarios combined with organic yields, group (b) includes the strongly heterogeneous BAU diets combined with relatively homogenous cropland yields (YIELDGAP), group (c) includes the same diet scenarios as (b), combined with regionally strongly divergent FAO yields, and group (d) includes the MEAT diet combined with the more homogenous YIELDGAP yields. Throughout the four scenario groups, the direction of trade flows for individual regions is very similar, but the volumes of the flows differ strongly. When looking at the medians, Europe, North America and Oceania, Russia and Central Asia, Latin America and South-East Asia are net exporters, whereas North Africa, Sub-Saharan Africa and South Asia are net importers. Only East Asia changes the direction of trade flows between the four scenario groups. Whereas Sub-Saharan Africa is a net importer of crop products, it is an exporter of ruminant products (expressed in roughage/grass equivalents). The latter can be explained by the vast grassland areas available in that region and the assumption in BioBaM that exported livestock products are produced in regions with large production potentials. South Asia, dominated by India, is relatively self-sufficient with regard to cropland products, with the exception of scenario group (d), but it is strongly dependent on imports of ruminant products. Note that the numbers shown in Fig. 5 represent the medians of scenario groups; extreme assumptions on factors such as cropland expansion can alter the magnitude and even the direction of trade flows in specific scenarios (Supplementary Table 9).

The first three scenario groups are comparable with regard to the global trade volumes, the fourth (MEAT diet combined with YIELDGAP yields) results in an almost twice as large global trade volume. In overall terms, a convergence of cropland yields (YIELDGAP) increases the self-sufficiency of developing countries, whereas converging diets decrease self-sufficiency.

## Discussion

Nearly two-thirds of all scenarios appear feasible or probably feasible in a world that—hypothetically—refrains from clearing any further forests for agricultural purposes. This result indicates that deforestation is not a precondition for supplying the world with sufficient food in terms of quantity and quality in 2050 and that many options exist based on different strategies. Our analysis reveals that even a global adoption of diets currently prevailing in the Western world would be feasible without deforestation if cropland yields rose massively and cropland expanded strongly into areas that are today used for grazing. Furthermore, high yields^17^ are no biophysical necessity; the world population can be fed healthily even with low cropland yields and little cropland expansion when diets with a reduced fraction of livestock products are adopted.

According to our analysis, human diets are the strongest determinant of the biophysical option space, stronger than yields or cropland availability. Unsurprisingly, vegan diets and diets with a low share of livestock products (for example, the VEGETARIAN variant) show the largest number of feasible scenarios, in line with other studies^19^,^33^,^40^, representing pathways that also make it possible to avoid the otherwise virulent grazing constraints and significantly reduce the option space. Other factors, such as high yields or intensive livestock systems, do not show such a strong effect on the number of feasible scenarios and do not necessarily reduce cropland demand or grazing intensity because the land-sparing effect can be annihilated by rich diets (Figs 3 and 4 and Supplementary Figs 3–6). These findings underpin the insight of other studies that stress the importance of demand-side measures for sustainability^26^,^27^,^33^,^41^. A vegan or vegetarian diet is associated with only half the cropland demand, grazing intensity and overall biomass harvest of comparable meat-based human diets. Furthermore, a decreasing share of livestock products in human diets could also be associated with health benefits, particularly in the industrialized regions^40^,^42^.

However, it is important to note that livestock provides many services other than food, for example, draught power, nutrient management and risk avoidance. For instance, livestock enables the use of land that cannot be used for cropping due to harsh environmental conditions and thus helps broaden society's resource base^33^,^34^,^43^. This effect becomes visible in our analysis in scenarios that combine low yields with little cropland expansion. In such contexts, diets relatively high in ruminant products show advantages over the monogastric-based variants. With increased cropland production, however, this advantage of ruminant livestock is lost.

Yields show a smaller effect than human diets on the overall option space, but low yield levels limit the number of feasible scenarios, particularly for diets with meat, which are affected primarily by cropland constraints. In this vein, our results suggest that even in a zero-deforestation world, low-yielding agriculture such as organic farming is a feasible option if paired with a vegetarian or vegan diet, or, to a lesser extent, if based on a massive cropland expansion, adding a nuanced perspective to this controversy^23^,^24^,^25^. In contrast, the expansion of cropland does not critically influence the option space, with the exception of the zero-cropland expansion variants, where approximately half of scenarios are not feasible. Cropland area and grazing intensity are strongly interlinked in our analysis. Under ‘zero deforestation,' large cropland entails smaller grazing lands and thus higher grazing intensity. The other factors assessed in our analysis do not show such strong overall effects within the entire option space, but they introduce variability within, for example, diet groups (Fig. 4 and Supplementary Fig. 7).

A further substantial contribution to widen the option space could be expected from reducing waste levels^8^. However, assessing the associated affects was beyond the scope of the paper, due to the intricacies of determining waste levels and discerning avoidable from unavoidable waste flows^44^. Therefore, we assumed low waste levels to prevail only in the four contract-converge scenarios (Supplementary Table 3). Consequently, the option space might be smaller if these low waste levels could not be reached. Climate-change effects on yields are not taken into account in this study, in line with the projections by the FAO^14^. Severe effects on the option space can be expected if yields are substantially decreased^45^. However, the effect of low yields on the option space is reflected in the ORGANIC variant in our assessment.

Our assessment reveals a particularly intricate trade-off related to food security. According to our scenarios, and in line with^19^, global dietary patterns that aim for an equal per capita provision of food (contract-converge scenarios) are, in general, terms bound to create trade-offs with targets of national self-sufficiency because they increase the import dependency of many developing regions. In regions with low purchasing power, a decrease in national self-sufficiency (*per se* neither necessary nor sufficient to guarantee food security at the individual level) may threaten food security^46^. We find that the reduction in self-sufficiency associated with the MEAT diet cannot, or can only partly, be compensated for by strategies that aim at ubiquitously closing currently prevailing yield gaps on cropland^19^, a strategy identified as instrumental to warranting food security and to reducing biomass harvest and cropland demand on the global scale^8^,^16^,^41^,^47^. Massive cropland expansion into grazing land could mitigate this trade-off (Supplementary Table 9). Our analysis reveals that this could reduce import dependencies in some regions, but it would do so at the expense of encroachment of farming into semi-natural or natural land, which is associated with considerable socio-ecological costs^48^. Note that our results do not assume any trade barriers because they were calculated as the biomass trade flows that would be required to compensate for regional deficits in biomass supply. Socioeconomic barriers or obstacles to biomass trade, which could result from subsidy systems, tariffs or other regulations, could narrow the options space by rendering more scenarios unfeasible. A better understanding of the conditions under which trade influences the development of agricultural productivity^49^,^50^ is hence a noteworthy scientific challenge.

Important constraints to the future option space result from limits to grazing intensity. Although cropland availability is a widely discussed planetary boundary^3^,^13^,^36^, many unknowns related to grazing limits prevail. This knowledge limitation is due primarily to the very limited data availability and the huge range of uncertainty related to the extent and intensity of grazing on the global scale^2^,^37^. In light of these data gaps, our results have been based on simple assumptions on grazing intensity thresholds and consistent data sets on land use and NPP patterns. There remains a lack of critical knowledge on, for example, the role of management, different livestock species and biomass flows and their geographic location but also on the interrelation between grazing and ecosystem processes and aspects of inter-annual variation (seasonality of grassland production). However, our finding that grazing pressure may become a prohibitive factor in many scenarios calls for concerted research in this area.

The option space analysed here is delineated solely on the basis of a biophysical balance between supply and demand. It is not aimed at exploring probabilities, and it does not support straightforward conclusions regarding the desirability, political practicability or sustainability performance of different scenarios. The approach enables exploration of the biophysical boundary conditions within which developments can unfold. Many more constraints and considerations become decisive when preferred solutions within this option space are to be identified. Assessment tools developed for such purposes need to weigh the full array of the direct and indirect costs^51^ and benefits of individual pathways to provide problem-shift robust results. A central trade-off relates to the area savings resulting from increased yields. These savings may increase carbon storage^52^,^53^, but this effect can potentially be compensated for by emissions from increased energy and resource demand in agriculture or increased biomass use^3^,^7^,^16^,^28^. The total amount of biomass required for the food system is important. For example, the benefits from increased soil carbon stocks of organic agriculture^22^ can be annihilated by the larger area demand resulting from lower yields of organic agriculture^54^. In this regard, the massive green-house gas emission costs associated with the expansion of cropland into grazing land, currently not well documented^48^,^55^, will be crucial. Analogous trade-offs can be suspected with topics such as nitrogen leaching, phosphorus depletion or biodiversity loss. In this context, scenarios that rely on smaller cropland areas and lower land use intensity levels could be favourable.

The identification of preferred future options would require additional analyses beyond biophysical analyses and the assessment of fundamental and complex economic, political and social effects associated with envisaged changes, such as the structural change in diet trajectories, farming practices, the replacement of land use systems and economic effects, for example, rising food prices.

Integrated assessment models enable assessment of the cost-benefit structures of future developments, often based on optimization approaches and conducted in detailed, economic sector-specific manner^50^. Complementary to such approaches, simple, transparent and data-based approaches such as those employed in this article enable scrutiny of the biophysical conditions, constraints and effects of anticipated changes in the land system, for example, by contextualizing results or by providing reality checks^56^. Fostering both research strands is a prerequisite for advancing our scientific understanding of the trade-offs related to land use and for identifying political strategies that allow developments to stay within the biophysical boundaries the Earth system poses to society.

## Methods

### Model framework and databases

The scenario analysis was performed with BioBaM, a biophysical accounting model that calculates the balance between biomass supply and biomass demand at the level of 11 world regions, for 14 biomass demand categories and corresponding primary commodities. BioBaM is based on consistent data on ecological and socioeconomic biomass flows and land use, and it respects thermodynamic principles (the law of conservation of mass and energy). It uses extensive databases for the year 2000, containing consistent data on socio-ecological biomass flows in ecosystems and socioeconomic systems (including, for example, NPP, used and unused harvests for 175 cultivars, the consumption of final products such as food and fibre, the differentiation of 11 final commodity groups), and it is consistent with spatially explicit information on land use^2^,^4^,^5^,^57^,^58^. Integrating these data sets into a model that allows consistent integration of biomass demand and supply flows, biophysical scenarios of the global agro-food system for 2050 were constructed, systematically combining four yield variants, five cropland expansion variants, two variants of the feedstuff composition of livestock diets, five human diet variants and three variants on the origin of livestock products on the demand side. For each of the resulting 500 scenarios, global biomass supply-demand balances were calculated for assessing the option space. The option space is defined as the sum of feasible scenarios—that is, when global demand for cropland products is matched by supply by at least by 95% (considering a 5% uncertainty range; cropland constraints) and livestock products' grazing intensity—that is, the ratio of grazed or mowed biomass to actually prevailing NPP^4^,^59^ stays below ecological thresholds (grazing constraints). In the absence of more reliable data, we assumed that no more than 70% of NPP could be grazed or mowed in highly productive grazing lands and that this ratio decreased with productivity, down to 25% in low-productive ecosystems such as steppes or semi-deserts^2^,^4^ (Supplementary Fig. 2), areas that are often under sporadic grazing regimes^2^,^4^,^37^. These maximum grazing intensities are far above the current levels. Feasibility is assessed on the global scale, and trade is assumed to balance regional differences in demand and supply, assuming no trade barriers prevail.

On the supply side, the model calculates (a) the potential supply of food and feed from cropland as a function of cropland availability and yield levels and (b) the potential roughage supply from grassland, calculated by combining estimates on available grazing land (remaining after cropland expansion) with estimates on actual NPP per unit area^4^ and the maximum achievable grazing intensities for four different grazing land classes, characterized by varying, region-specific maximum grazing suitability^2^,^4^. Areas for cropland and grazing land are taken from Erb *et al.*^2^.

On the demand side, the model calculates for each specific human diet (a) the demand of primary crops for food and feed from cropland and (b) roughage demand for the production of meat and milk from grassland (see below). It discerns 14 product groups, for example, cereals, pulses, ruminant meat and eggs. The per capita food demand was multiplied by total population numbers^60^, plus an added fraction for household food waste^61^. We converted household crop demand to primary crop demand by applying region- and crop-specific (a) seed factors and b) factors for processing losses, wastes and byproducts (for example, brans in flour production, based on commodity balances by^62^). All biomass data were converted into the unit dry biomass based on water-content tables^57^.

Regional deficits in crop (both food and feed) or roughage supply is assumed to be compensated for by interregional trade. The volume of regional net trade was assessed the following way: for all feasible scenarios, the deficit of crop products (both food and feed) or roughage in a region was assumed to be compensated for by a surplus production of crop products and roughage in those regions with highest remaining production potentials after subtracting domestic consumption. Thus, we present a biophysical net trade balance that does not include, for example, any economically induced trade barriers. To yield comparable results, we express trade flows of ruminant products in roughage equivalents, that is, the amount of roughage that would be required to close the regional supply deficit of meat and milk product demand.

### Parameters and variants

Four yield variants are calculated: HIGH, FAO, YIELDGAP and ORGANIC, all variants discerning 11 crop groups. FAO denotes yields of the FAO projection^14^ and serves as starting point. These projections are available only for some crops and have been complemented based on alternative data sets. The HIGH variant is in line with the Global Orchestration scenario by the Millennium Ecosystem Assessment^63^ and is 9% above the FAO variant. The YIELDGAP variant assumes the yield gap to be closed to an attainable maximum, resulting in a less heterogeneous global pattern of cropland yields^47^. The ORGANIC variant assumes lower yields in industrialized systems and reflects yield losses due to organic farming over business-as-usual trajectories^25^,^64^,^65^. Note that in this variant, regions with little industrialized agriculture are not affected by yield reductions (Supplementary Table 6).

On the basis of the literature and our own assessments, five variants of cropland expansion were constructed (+0%, +11%, +22%, +40% and +70%). For all these variants, it was assumed that cropland expands only into grazing land of the highest productivity. The +11% variant is in line with the FAO projection^14^ and results in a cropland expansion by 11% over the year 2000. In the +22% variant, this value is doubled in each region. Variants +40 and +70% assume that 50 and 100%, respectively, of the highly productive grazing lands are converted into cropland, resulting in a global expansion of cropland over 2000 by 40 and 70%. Regional cropland expansion varies largely for all scenarios (Supplementary Table 7). As a consequence of cropland expansion, the grazing area is assumed to shrink to the same extent. We did not assess variants of cropland expansion into grazing land of lower productivity, such as savannas or steppes, or into wilderness areas because of the high costs (for example, infrastructure demand) associated with such a strategy and the apparent difficulties to achieve similar crop-yield levels.

Regional estimates of feedstuff composition and feeding-conversion ratios are based on linear extrapolations of the trajectories between 1995 and 2030 provided by Bouwman *et al.*^66^ The data refer to two feed categories, crop-based feed (for example, cereals and oil cake) and roughage (straw and grass). The variant GRAIN assumes a 30% increase in crop-based feeds in feedstuff composition, whereas ROUGH reduces crop-based feed in feedstuff composition by 50% in all regions. Differences in the nutritional values of grain-based and roughage-based feedstuffs were taken into account based on data taken from^57^,^66^.

Five human diets are discerned, all providing sufficient energy and protein. The BAU variant is in line with FAO^14^ and yields a global average diet of 2,947 kcal per cap per day with large regional differences. In the RICH variant, the per capita food demand of all regions in 2050 converges to per capita food demand of North America in 2000 (ref. 62), yielding 3.546 kcal per cap per day. The variants MEAT (a reduced meat diet), VEGETARIAN (ovo-lacto vegetarian) and VEGAN (exclusively plant-based) follow USDA recommendations^67^ and are characterized with a per capita intake of 2,648 for MEAT and 2,636 kcal per cap per day for VEGETARIAN and VEGAN in all regions. This value is close to the global average in the year 2000 (2,657 kcal per cap per day) (ref. 62). The share and type of livestock-product composition varies, being 25% in the MEAT diet, 13% in the VEGETARIAN diet (only milk and eggs) and 0% in the VEGAN diet (Supplementary Table 2). The global demand for livestock products in the MEAT variant is 50% higher than that in the global average of the BAU variant, but it is 26–36% lower than the BAU demand in Western Europe and North America, respectively.

For each human diet scenario, we assumed different origins of livestock products. The BAU variant is based on the respective regional diet variant according to FAO projections. The MONOGASTRIC variant assumes all livestock products originate from monogastric species (eggs, in the case of the vegetarian diet), and the opposite is assumed for the RUMINANT variant (milk, in the case of the vegetarian diet).

## Additional information

**How to cite this article:** Erb, K.-H. *et al.* Exploring the biophysical option space for feeding the world without deforestation. *Nat. Commun.* 7:11382 doi: 10.1038/ncomms11382 (2016).

## Supplementary Material

Supplementary Information Supplementary Figures 1-7, Supplementary Tables 1-9, Supplementary Methods and Supplementary References

## Figures and Tables

**Figure 1:**
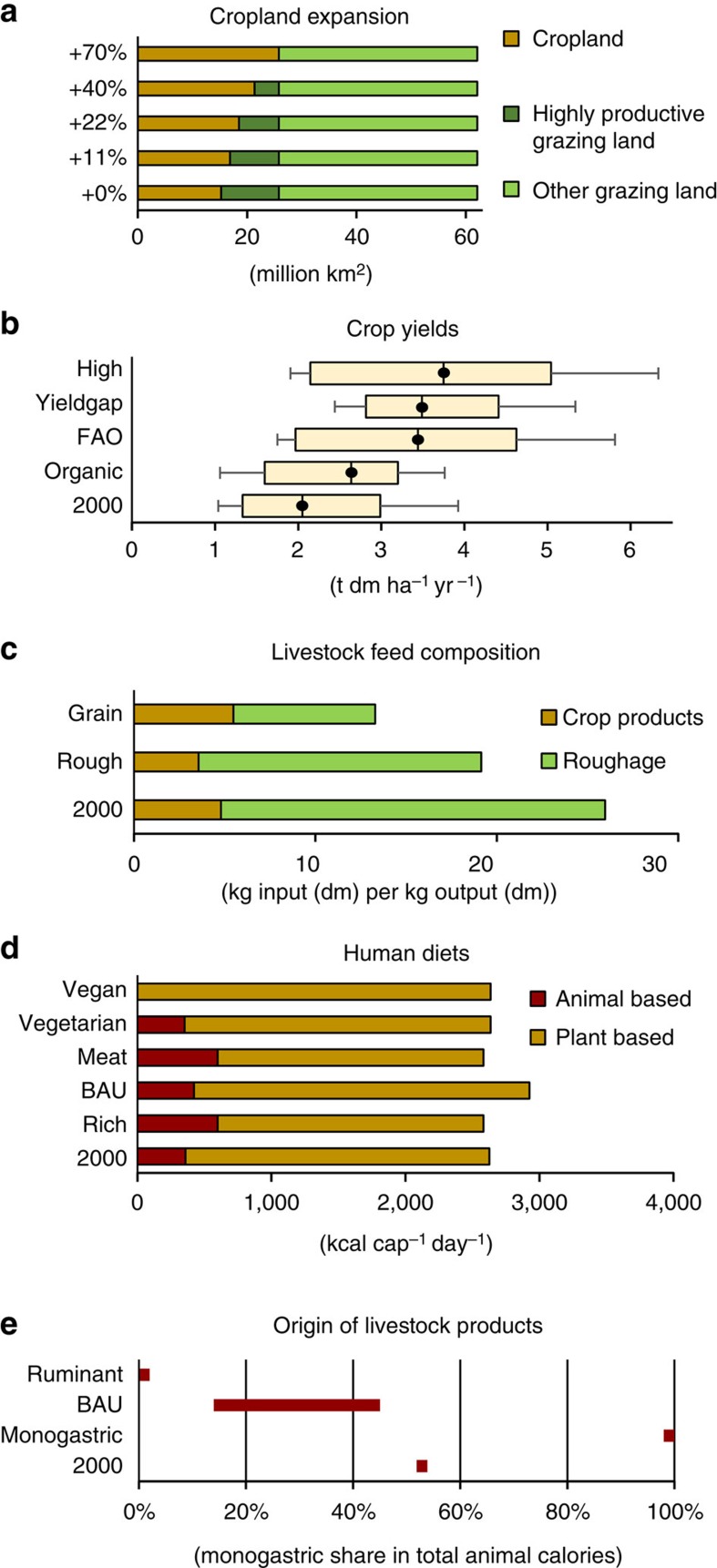
Key parameters and their variants combined within BioBaM and values for the year 2000. (**a**) Cropland expansion variants (million km^2^), from no-expansion (+0%) to full expansion into highly productive grazing land^2^ (+70%). The +11% variant is in line with FAO forecasts^14^. (**b**) Boxplots of the region- and cultivar-specific crop yields in each variant (t dm ha^−1^ yr^−1^), from extensive low organic yield to intensive-farming, high-yield variants. Coloured boxes indicate the two inner quartiles (>25 and <75) per world region; whiskers indicate the minimum and maximum values. (**c**) Variants of livestock feedstuff composition based on feed-conversion ratios (kg input (dm) per kg output (dm)). (**d**) Variant of human diets, quantity and composition (kcal per cap per day). VEGAN denotes a diet without livestock products; VEGETARIAN denotes a diet without meat but with eggs, and milk. These two and MEAT, a diet with a considerable fraction of livestock meat, are literature derived^67^ and represent recommendations based on health considerations. The BAU scenario is in line with the FAO forecast for 2050 (ref. 14), whereas the RICH scenario assumes the diet of North America in 2000 to prevail globally in 2050. All variants except 2000 and BAU assume a global convergence of per capita diets. (**e**) Variants of the origin of meat in human diet variants expressed as monogastric share in total animal calories. The bar for the BAU variant refers to the range of all diet variants with the exception of VEGAN. The combination of all variants results in 500 individual scenarios. (Note that the VEGAN diet variant does not include variants of livestock system parameters (**c**,**e**)). Values for 2000 from (refs 57, 66).
