# Supplementary material for: Exploring the biophysical option space for feeding the world without deforestation
Source: Nat Commun. 2016 Apr 19;7:11382. doi: 10.1038/ncomms11382 (PMC4838894; doi:10.1038/ncomms11382)
Supplement: Supplementary Information — Supplementary Figures 1-7, Supplementary Tables 1-9, Supplementary Methods and Supplementary References [file ncomms11382-s1.pdf]

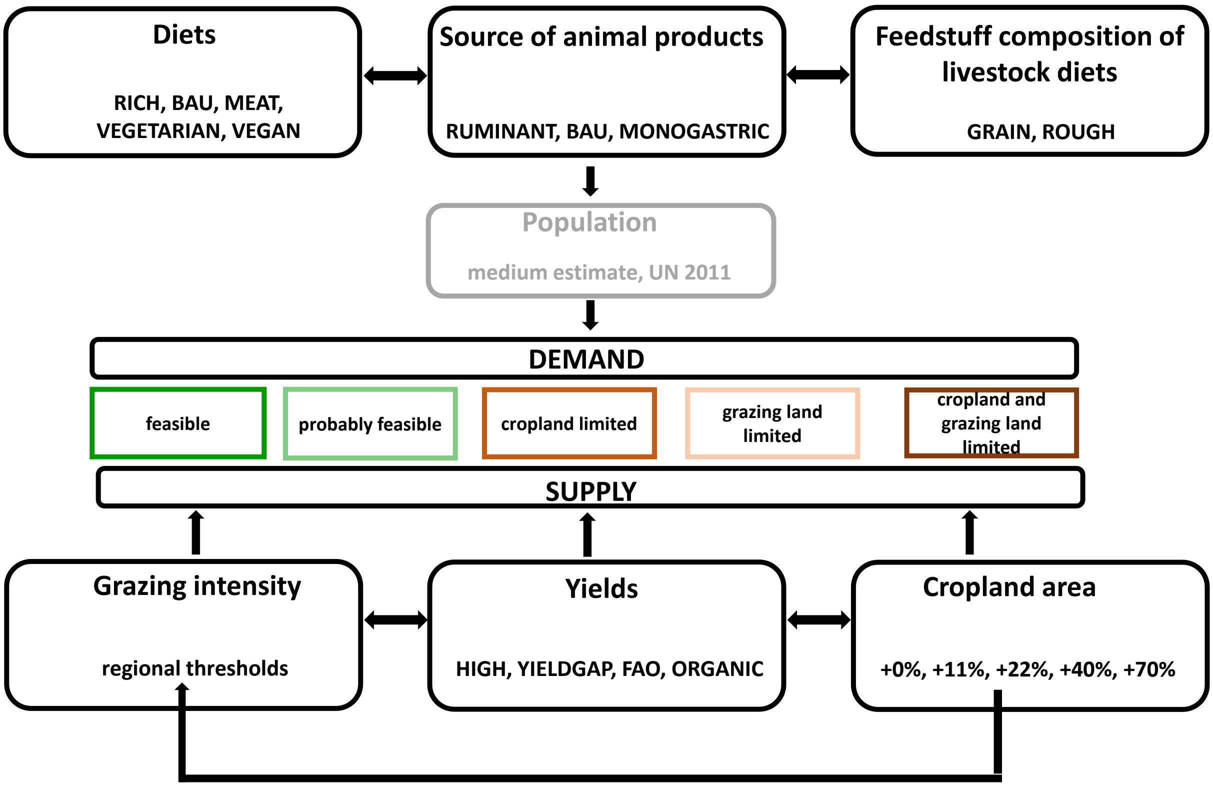

Supplementary Figure 1. Schematic representation of the BioBaM model.

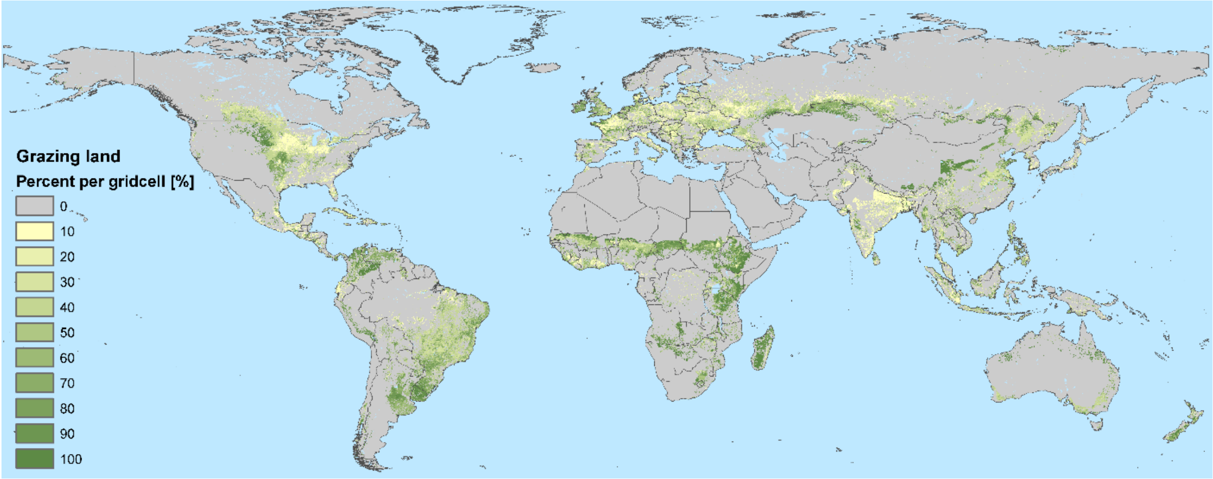

Supplementary Figure 2. Extent of grazing land potentially available for the various variants of cropland expansion. Cropland is assumed to only expand into these areas, which are identified by remote sensing[1] as being under management and are characterized by a productivity threshold[2] (>20gC m^{-2} yr^{-1} aboveground net primary production).

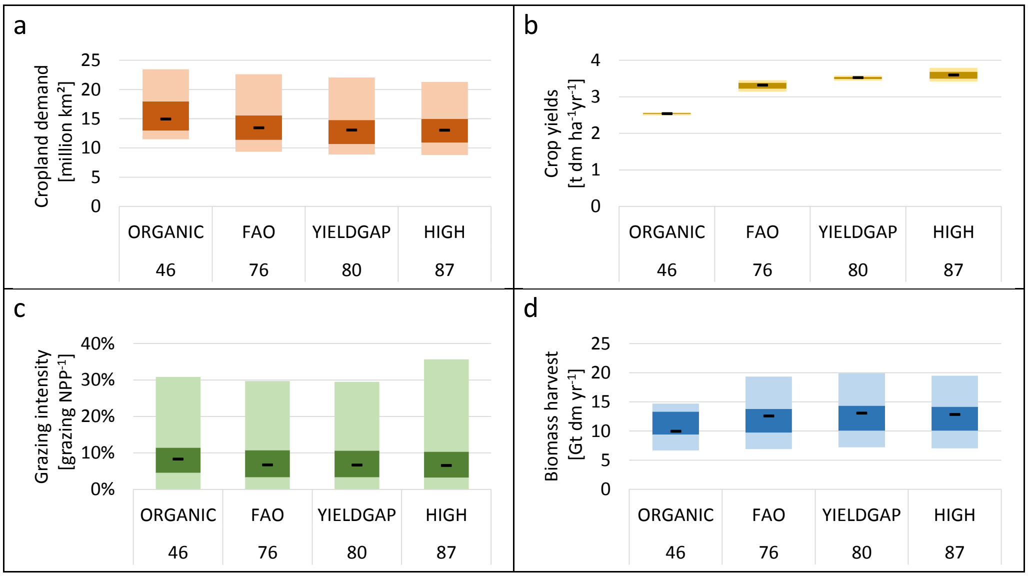

Supplementary Figure 3. Characterization of feasible and probably feasible scenarios, breakdown to crop yields. a) Cropland demand, b) average cropland yields, c) grazing intensity, d) ratio of total harvest to total potential NPP on agricultural areas. Dark boxes indicate the two inner quartiles (>25 and <75%) of all feasible scenarios, light colour-shaded boxes the minimum and maximum values. Small lines indicate the median. Numbers below diets indicate the number of feasible scenarios.

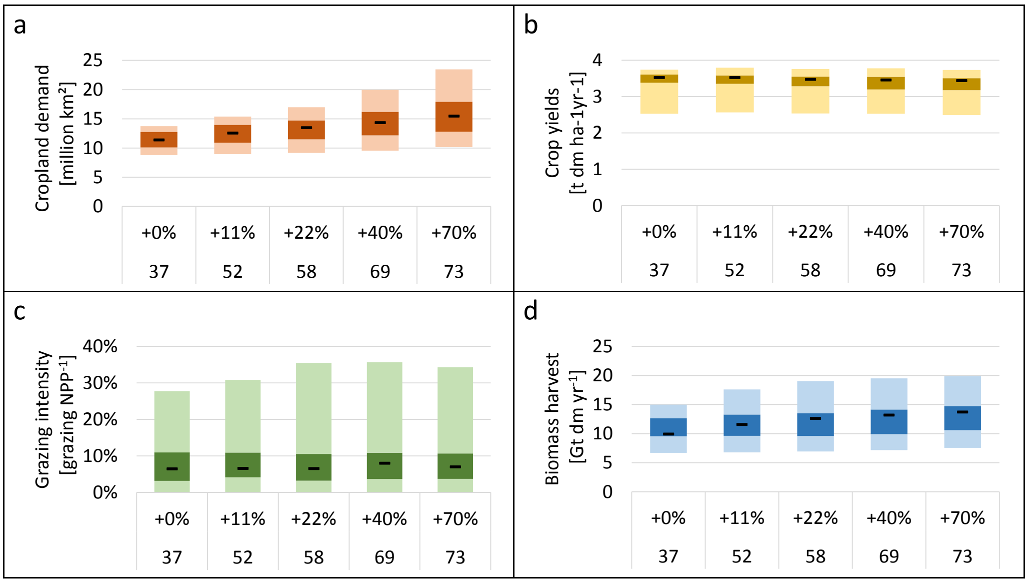

Supplementary Figure 4. Characterization of feasible and probably feasible scenarios, breakdown to cropland expansion. a) Cropland demand, b) average cropland yields, c) grazing intensity, d) ratio of total harvest to total potential NPP on agricultural areas. Dark boxes indicate the two inner quartiles (>25 and <75%) of all feasible scenarios, light colour-shaded boxes the minimum and maximum values. Small lines indicate the median. Numbers below diets indicate the number of feasible scenarios.

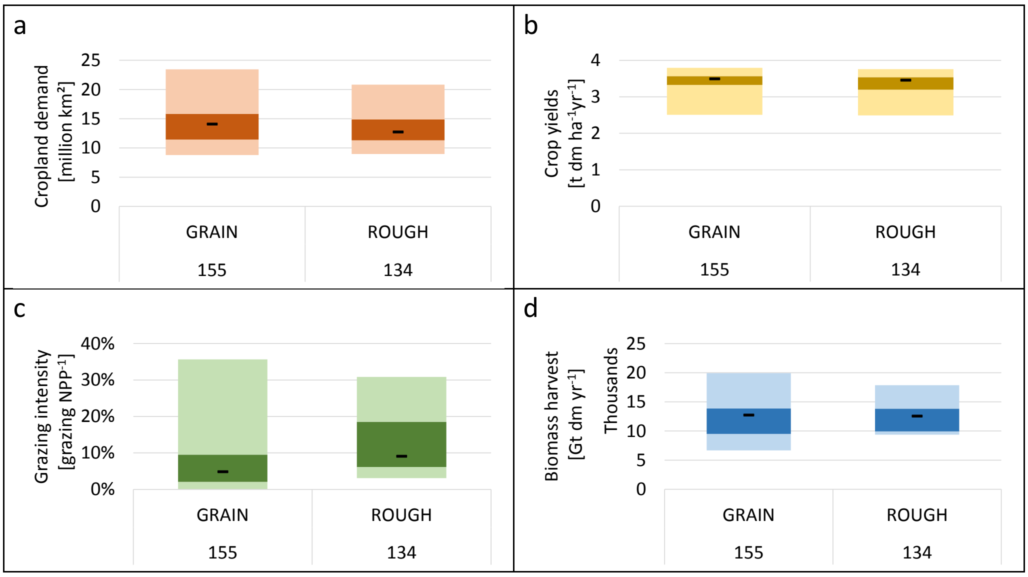

Supplementary Figure 5. Characterization of feasible and probably feasible scenarios, breakdown to feedstuff composition. a) Cropland demand, b) average cropland yields, c) grazing intensity, d) ratio of total harvest to total potential NPP on agricultural areas. Dark boxes indicate the two inner quartiles (>25 and <75%) of all feasible scenarios, light colour-shaded boxes the minimum and maximum values. Small lines indicate the median. Numbers below diets indicate the number of feasible scenarios.

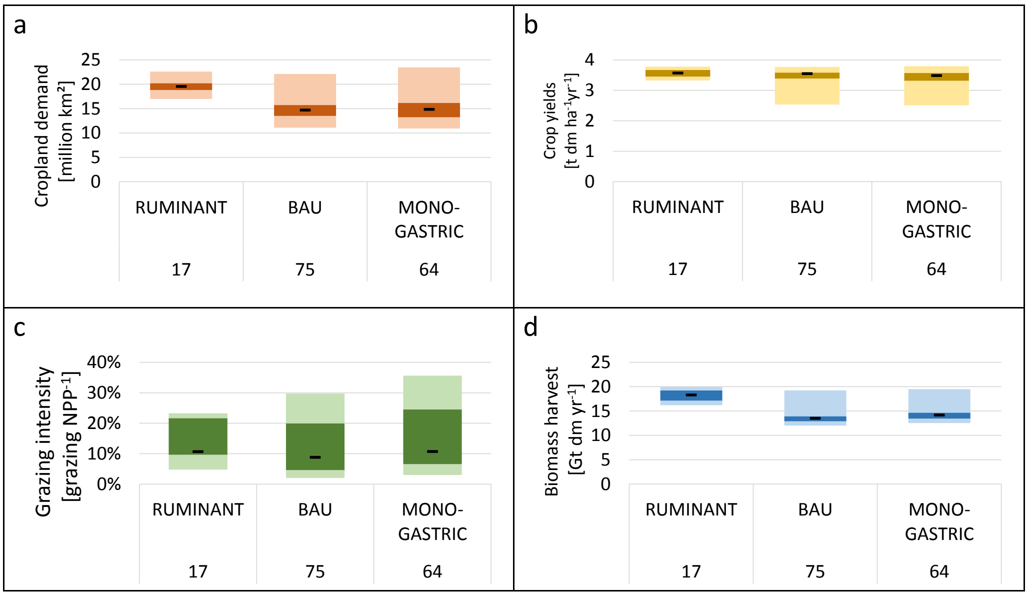

Supplementary Figure 6. Characterization of feasible and probably feasible scenarios, breakdown to source of livestock products in human diets. a) Cropland demand, b) average cropland yields, c) grazing intensity, d) ratio of total harvest to total potential NPP on agricultural areas. Dark boxes indicate the two inner quartiles (>25 and <75%) of all feasible scenarios, light colour-shaded boxes the minimum and maximum values. Small lines indicate the median. Numbers below diets indicate the number of feasible scenarios..

			Human diets: RICH			BAU			MEAT			VEGETARIAN			VEGAN*
	Cropland expansion	feed basis	Source of animal products: ruminant	bau	monogastric	ruminant	bau	monogastric	ruminant	bau	monogastric	ruminant	bau	monogastric	
high yield	+70%	rough			17.2		14.2	13.4		14.9	14.1	10.2	10.4	11.6	8.2
high yield	+70%	grain		19.4	18.5	18.9	13.7	14.0	19.5	13.9	15.0	10.1	10.4	12.1	8.2
high yield	+40%	rough			16.6		13.7	12.8		14.6	13.4	9.7	9.9	10.9	7.7
high yield	+40%	grain		19.1	17.9	18.3	13.2	13.5	19.3	13.5	14.4	9.5	9.8	11.4	7.7
high yield	+22%	rough			16.2		13.5	12.4		14.4	13.1	9.4	9.6	10.6	7.4
high yield	+22%	grain				17.9	12.9	13.1	19.0	13.2	14.0	9.2	9.6	11.0	7.4
high yield	+11%	rough					13.5	12.2		14.6	12.7	9.8	9.8	10.3	7.2
high yield	+11%	grain				17.6	12.7	12.8		13.2	13.7	9.4	9.6	10.8	7.2
high yield	+0%	rough					13.6	12.0		14.7	12.5	9.7	9.8	10.1	7.0
high yield	+0%	grain					12.6			13.3		9.3	9.5	10.6	7.0
yield gap closed	+70%	rough			17.9		14.5	13.8		15.1	14.6	10.4	10.6	11.9	8.5
yield gap closed	+70%	grain		19.9	19.2	19.3	14.1	14.5		14.3	15.6	10.3	10.6	12.4	8.5
yield gap closed	+40%	rough			17.1		14.0	13.2		14.8	13.9	9.8	10.1	11.3	7.9
yield gap closed	+40%	grain		19.5		18.4	13.6	13.8		13.8	14.9	9.7	10.1	11.8	7.9
yield gap closed	+22%	rough					13.8	12.8		14.6	13.5	9.5	9.7	10.9	7.6
yield gap closed	+22%	grain				17.8	13.2	13.4		13.5	14.4	9.3	9.7	11.4	7.6
yield gap closed	+11%	rough					13.8	12.4		14.7	13.1	9.5	9.7	10.6	7.4
yield gap closed	+11%	grain				17.6	13.0	13.0		13.5		9.4	9.7	11.1	7.4
yield gap closed	+0%	rough					13.8	12.2		15.0		9.8	9.9	10.3	7.2
yield gap closed	+0%	grain					13.0			13.6		9.5	9.7	10.9	7.2
FAO yield	+70%	rough			17.0		14.0	13.1		14.9	13.8	10.1	10.2	11.3	8.0
FAO yield	+70%	grain		19.3	18.3	18.6	13.5	13.8		13.8	14.8	9.9	10.2	11.8	8.0
FAO yield	+40%	rough			16.3		13.6	12.6		14.6	13.2	9.6	9.7	10.7	7.5
FAO yield	+40%	grain		19.1		18.0	13.1	13.3		13.5	14.1	9.4	9.7	11.2	7.5
FAO yield	+22%	rough					13.5	12.3		14.5	12.8	9.4	9.7	10.4	7.2
FAO yield	+22%	grain				17.7	12.8	12.8		13.2	13.8	9.3	9.6	10.8	7.2
FAO yield	+11%	rough					13.5	12.0		14.6	12.5	9.7	9.7	10.1	7.0
FAO yield	+11%	grain				17.4	12.6			13.2		9.3	9.5	10.6	7.0
FAO yield	+0%	rough					13.6			14.8		9.7	9.7	9.9	6.9
FAO yield	+0%	grain										9.2	9.5	10.5	6.9
organic yields	+70%	rough					13.7	12.7		14.7	13.3	9.7	9.8	10.8	7.6
organic yields	+70%	grain					13.2	13.3		13.5	14.3	9.4	9.7	11.3	7.6
organic yields	+40%	rough					13.5	12.1		14.6	12.8	9.4	9.5	10.3	7.2
organic yields	+40%	grain					12.8			13.3		9.0	9.3	10.8	7.2
organic yields	+22%	rough					13.5			14.4		9.5	9.6	10.0	6.9
organic yields	+22%	grain										9.2	9.4		6.9
organic yields	+11%	rough					13.5			14.7		9.6	9.6		6.8
organic yields	+11%	grain										9.1	9.3		6.8
organic yields	+0%	rough										9.6	9.8		6.7
organic yields	+0%	grain													6.7

Supplementary Figure 7. Biomass harvest of feasible and probably feasible scenarios. Values indicate total biomass harvest volumes on agricultural area in Mtdm yr^{-1}. Green colors indicate low values, red colors high values. *for the VEGAN diet variant, the source of livestock products is not relevant.

Supplementary Table 1. Household food demand in 2000 for all regions in kcal cap^{-1} day^{-1} according to FAOSTAT. Includes household food waste (Supplementary Table 3).

	2000											
	NAWA	SSAfrica	CARussia	EAsia	SAsia	SEAsia	NAmerica	LAmerica	WEurope	EEurope	Oceania	Global avg.
Cereals	1 731	1 072	1 290	1 601	1 426	1 551	1 014	1 104	1 017	1 263	820	1 368
Roots	70	462	183	169	47	92	109	141	140	192	117	154
Sugarcrops	301	105	326	90	233	192	613	405	385	358	413	236
Pulses	70	89	8	15	96	25	42	105	32	25	19	56
Oilcrops	323	234	233	258	213	232	626	326	498	278	464	287
Vegetables and fruits	221	64	110	187	91	105	214	144	271	160	218	145
Other crops	34	17	16	12	23	15	45	17	59	24	30	22
Meat (ruminants)	59	38	110	36	16	14	121	111	92	55	229	49
Pigs, poultry, eggs	70	28	123	392	15	120	365	193	370	240	233	189
Milk, butter, dairy	131	50	250	29	109	21	367	172	327	266	266	120
Fish	16	14	29	54	9	48	31	19	54	20	35	31
**Total kcal/cap/day**	**3 027**	**2 171**	**2 678**	**2 842**	**2 278**	**2 413**	**3 546**	**2 736**	**3 246**	**2 881**	**2 845**	**2 657**
**Share of animal products**	**9%**	**6%**	**19%**	**18%**	**7%**	**8%**	**25%**	**18%**	**26%**	**20%**	**27%**	**15%**

Supplementary Table 2. Region specific household food demand and global average in 2050, given in kcal $cap^{-1}day^{-1}$. Includes household food waste (Supplementary Table 3). The variant BAU is based on FAO projections [3]. For the variants MEAT, RICH, VEGETARIAN, and VEGAN we assume converging regional diets that arrive at the same values for each region in 2050. See Supplementary Methods for further explanations.

	2050															
	BAU												RICH	MEAT	VEGETARIAN	VEGAN
	NAWA	SSAfrica	CARussia	EAsia	SAsia	SEAsia	NAmerica	LAmerica	WEurope	EEurope	Oceania	Global avg.	all regions	all regions	all regions	all regions
Cereals	1 537	1 392	1 314	1 478	1 403	1 422	1 032	1 191	1 035	1 286	676	1 363	1 013	846	836	844
Roots	73	441	169	135	79	73	101	142	130	178	87	176	109	189	188	189
Sugarcrops	294	167	321	149	258	318	601	401	378	351	328	265	612	173	168	169
Pulses	57	119	8	13	86	22	43	97	33	26	16	67	42	73	169	215
Oilcrops	354	362	272	454	411	404	731	407	581	325	439	422	626	279	364	628
Vegetables and fruits	274	89	160	278	134	155	309	202	393	232	263	188	214	357	352	355
Other crops	31	17	16	12	23	14	45	17	59	24	24	22	45	67	207	235
Meat (ruminants)	93	40	60	122	19	24	140	144	120	85	76	69	121	61	0	0
Pigs, poultry, eggs	88	37	164	519	82	104	385	246	329	232	323	191	365	238	49	0
Milk, butter, dairy	160	65	284	98	189	70	415	216	370	301	244	162	367	300	303	0
Fish	36	35	56	126	22	110	59	47	106	38	50	59	31	65	0	0
**Total kcal/cap/day**	**2 996**	**2 763**	**2 822**	**3 387**	**2 707**	**2 717**	**3 861**	**3 108**	**3 534**	**3 077**	**2 526**	**2 983**	**3 545**	**2 648**	**2 636**	**2 636**
**Share of animal products**	**13%**	**6%**	**20%**	**26%**	**12%**	**11%**	**26%**	**21%**	**26%**	**21%**	**27%**	**16%**	**25%**	**25%**	**13%**	**0%**

Supplementary Table 3. Region specific household food waste and global average in 2000, given as percentage of household food demand in kcal, based on Gustavsson et al. [4]. See Supplementary Methods for further explanations.

	NAWA	SSAfrica	CARussia	EAsia	SAsia	SEAsia	NAmerica	LAmerica	WEurope	EEurope	Oceania	Global avg.
Cereals	12%	1%	25%	20%	3%	3%	27%	10%	25%	25%	27%	12%
Roots	6%	2%	17%	10%	3%	3%	30%	4%	17%	17%	30%	8%
Sugarcrops	2%	1%	4%	4%	1%	1%	4%	2%	4%	4%	4%	3%
Pulses	2%	1%	4%	4%	1%	1%	4%	2%	4%	4%	4%	2%
Oilcrops	2%	1%	4%	4%	1%	1%	4%	2%	4%	4%	4%	3%
Vegetables and fruits	12%	5%	19%	15%	7%	7%	28%	10%	19%	19%	28%	14%
Other crops	2%	1%	4%	4%	1%	1%	4%	2%	4%	4%	4%	8%
Meat (ruminants)	8%	2%	11%	8%	4%	4%	11%	6%	11%	11%	11%	6%
Pigs, poultry, eggs	8%	2%	11%	8%	4%	4%	11%	6%	11%	11%	11%	8%
Milk, butter, dairy	2%	0%	7%	5%	1%	1%	15%	4%	7%	7%	15%	3%
Fish	4%	2%	11%	8%	2%	2%	33%	4%	11%	11%	33%	9%

Supplementary Table 4. Input-output ratios between feed input and livestock product output for 2000 and the two variants GRAIN and ROUGH in 2050. All values are given in tons of feed input (dry matter) per ton of output (dry matter).

		**2000**										
		**NAWA**	**SSAfrica**	**CARussia**	**EAsia**	**SAsia**	**SEAsia**	**NAmerica**	**LAmerica**	**WEurope**	**EEurope**	**Oceania**
Feed crops	Meat (ruminants)	4.0	2.1	4.6	2.7	3.3	3.3	3.3	2.0	4.7	4.7	3.7
	Pigs, poultry, eggs	7.1	8.4	7.2	7.2	8.5	7.2	6.8	7.3	7.0	6.9	6.8
	Milk, butter, dairy	4.0	2.1	4.6	2.7	3.3	3.3	3.3	2.0	4.7	4.7	3.7
Roughage feed	Meat (ruminants)	21.0	90.9	12.6	45.5	35.9	51.0	32.7	57.1	15.4	10.6	29.9
	Pigs, poultry, eggs	-	-	-	-	-	-	-	-	-	-	-
	Milk, butter, dairy	21.0	90.9	12.6	45.5	35.9	51.0	32.7	57.1	15.4	10.6	29.9
		**2050**										
		**GRAIN**										
Feed crops	Meat (ruminants)	7.0	5.0	7.7	11.8	5.0	8.4	16.7	5.0	5.0	11.1	5.0
	Pigs, poultry, eggs	8.0	6.5	8.0	5.2	5.8	5.1	8.0	7.5	8.0	8.0	8.0
	Milk, butter, dairy	3.0	1.2	2.6	4.0	1.0	1.1	3.9	2.3	1.8	3.6	1.0
Roughage feed	Meat (ruminants)	26.3	30.0	30.0	30.0	30.0	30.0	30.0	30.0	13.0	30.0	30.0
	Pigs, poultry, eggs	0.5	2.5	2.3	4.1	1.9	4.1	0.5	0.6	1.5	2.8	0.5
	Milk, butter, dairy	5.9	10.0	5.8	7.9	10.0	7.5	2.5	10.0	7.1	4.3	7.1
		**ROUGH**										
Feed crops	Meat (ruminants)	2.7	0.4	3.0	4.5	0.7	3.2	6.4	1.8	1.1	4.3	0.4
	Pigs, poultry, eggs	7.1	5.0	7.7	4.0	4.4	3.9	6.4	5.8	6.8	7.8	6.2
	Milk, butter, dairy	1.2	0.5	1.0	1.5	0.3	0.4	1.5	0.9	0.7	1.4	0.2
Roughage feed	Meat (ruminants)	34.9	71.8	41.6	61.5	70.0	95.4	50.9	97.9	20.8	51.4	60.7
	Pigs, poultry, eggs	1.8	5.5	3.0	6.5	4.6	6.5	3.1	4.1	3.9	3.2	3.8
	Milk, butter, dairy	9.6	23.1	9.0	12.8	17.3	8.9	7.3	19.8	9.3	8.7	8.8

Supplementary Table 5. Grazing classes and assumed grazing intensity thresholds. These intensity thresholds follow the assumption by [5]. For all regions, grazing intensity beyond these thresholds or triple the actual grazing intensity have been assumed to be unfeasible, with the exception of South Asia. Here, no grazing intensity thresholds have been employed, because in the year 2000 levels already exceed 100% due to statistical uncertainties [5].

**Grazing class**	**10^6 km² (% of total)**	**Land cover class (GLC2000)**	**Aboveground Productivity (LPJ-DGVM)****	**Current grazing intensity*~**	**Intensity threshold***
Grazing class 1	11.4 (24%)	16: Cultivated and managed areas<br>13: Herbaceous cover, closed-open	>200gC $m^{-2}yr^{-1}$	46%	70%
Grazing class 2	7.4 (16%)	1–8: Tree cover eight classes,<br>9: Mosaic tree cover/natural vegetation<br>10: Tree cover, burnt (mainly boreal forests)<br>15: Regularly flooded shrub and/or herbaceous cover<br>20: Water bodies (included for reasons of completeness)<br>22: Urban areas (included for reasons of completeness)	>200gC $m^{2-1}yr^{-1}$	5%	55%
Grazing class 3	8.2 (18%)	1–8: Tree cover eight classes,<br>9: Mosaic tree cover/natural vegetation<br>10: Tree cover, burnt (mainly boreal forests)<br>15: Regularly flooded shrub and/or herbaceous cover<br>16: Cultivated and managed areas<br>13: Herbaceous cover, closed-open<br>20: Water bodies (included for reasons of completeness)<br>22: Urban areas (included for reasons of completeness)	<200gC $m^{2-1}yr^{-1}$	15%	40%
Grazing class 4	19.9 (42%)	11: Shrub cover, closed to open evergreen (broadleaved or needle leaved)<br>12: Shrub cover, closed to open deciduous (broadleaved)<br>14: Sparse herbaceous or sparse shrub cover<br>19: Bare areas<br>21: Snow or ice (included for reasons of completeness).		13%	25%

* measured as ratio of harvest to aboveground actual NPP

~ year 2000, data from [5]

**LPJ-DGVM (Lund-Potsdam-Jena Dynamic Global Vegetation Model) results taken from[5].

Supplementary Table 6. Region specific crop yields in tons of dry matter per hectare (t dm^{-1} ha^{-1}) in 2000 and for all variants in 2050.

	NAWA	SSAfrica	CARussia	EAsia	SAsia	SEAsia	NAmerica	LAmerica	WEurope	EEurope	Oceania
**2000**											
Cereals	1.28	0.89	1.46	4.52	2.15	2.91	3.27	1.91	4.60	1.48	1.41
Roots	3.01	2.19	2.94	5.19	4.16	3.63	6.07	2.60	7.36	1.38	2.74
Sugarcrops	8.88	9.07	7.01	11.20	11.57	10.47	9.25	8.78	12.92	3.10	12.34
Pulses	0.64	0.42	1.67	1.34	0.56	0.70	1.16	0.52	2.40	0.56	0.79
Oilcrops	0.51	0.87	1.02	1.84	0.97	3.66	1.59	1.69	1.58	0.66	1.30
Vegetables and fruits	1.40	1.19	1.00	1.52	1.67	1.34	2.37	1.76	1.83	0.63	1.97
Other crops	1.27	0.50	1.35	2.37	0.94	0.52	1.32	0.66	1.38	0.65	1.98
**2050**											
**HIGH**											
Cereals	2.21	2.15	1.95	6.36	3.83	4.47	5.90	3.47	7.82	2.18	1.88
Roots	5.37	3.51	4.37	7.91	7.51	6.26	9.93	3.53	11.35	1.37	3.70
Sugarcrops	12.96	17.58	11.61	14.55	18.35	12.31	11.38	14.04	15.59	3.94	14.58
Pulses	0.88	0.85	2.67	1.92	1.22	1.17	1.67	0.70	3.32	1.57	0.94
Oilcrops	1.38	2.12	2.52	4.33	3.13	9.48	3.80	4.18	2.82	1.10	2.11
Vegetables and fruits	2.28	1.58	1.66	1.79	2.74	2.28	3.48	2.33	2.61	0.68	2.77
Other crops	2.08	0.76	2.32	2.59	1.43	0.86	1.78	1.04	0.81	0.55	2.80
**YIELDGAP**											
Cereals	3.20	2.61	3.41	6.11	3.93	4.41	4.20	3.83	5.12	3.40	2.64
Roots	6.03	4.60	8.18	9.08	6.75	5.14	7.58	4.81	8.66	2.98	3.05
Sugarcrops	14.55	18.60	20.16	15.35	18.69	17.39	11.62	13.73	14.78	6.73	14.47
Pulses	0.81	0.78	2.45	1.77	1.12	1.07	1.53	0.65	3.05	1.44	0.86
Oilcrops	0.57	1.95	2.40	2.55	1.63	6.33	2.10	2.20	1.85	1.06	1.94
Vegetables and Fruits	2.09	1.45	1.52	1.64	2.52	2.09	3.19	2.13	2.40	0.63	2.54
Other crops	1.91	0.70	2.13	2.38	1.32	0.79	1.63	0.95	0.75	0.51	2.56
**FAO**											
Cereals	2.03	1.97	1.79	5.84	3.51	4.10	5.41	3.18	7.17	2.00	1.72
Roots	4.93	3.22	4.01	7.26	6.89	5.75	9.11	3.24	10.41	1.25	3.40
Sugarcrops	11.89	16.13	10.65	13.35	16.84	11.30	10.44	12.88	14.30	3.61	13.38
Pulses	0.81	0.78	2.45	1.77	1.12	1.07	1.53	0.65	3.05	1.44	0.86
Oilcrops	1.27	1.94	2.31	3.97	2.87	8.69	3.48	3.84	2.59	1.01	1.93
Vegetables and Fruits	2.09	1.45	1.52	1.64	2.52	2.09	3.19	2.13	2.40	0.63	2.54
Other crops	1.91	0.70	2.13	2.38	1.32	0.79	1.63	0.95	0.75	0.51	2.56
**ORGANIC**											
Cereals	1.55	1.89	1.43	3.74	3.23	3.28	3.26	2.24	4.43	1.21	1.40
Roots	3.75	3.09	3.21	4.66	6.35	4.60	5.49	2.27	6.44	0.76	2.76
Sugarcrops	9.05	15.49	8.52	8.56	15.51	9.04	6.29	9.05	8.84	2.19	10.85
Pulses	0.62	0.75	1.96	1.13	1.03	0.86	0.92	0.45	1.89	0.87	0.70
Oilcrops	0.96	1.87	1.85	2.55	2.64	6.96	2.10	2.70	1.60	0.61	1.57
Vegetables and fruits	1.59	1.39	1.22	1.05	2.32	1.68	1.92	1.50	1.48	0.38	2.06
Other crops	1.45	0.67	1.70	1.52	1.21	0.63	0.98	0.67	0.46	0.31	2.08

Supplementary Table 7. Region and crop specific cropland areas for the year 2000, and the different cropland expansion variants +11%, +22%, +40% and +70%. All values are given in million hectares (Mha). The variant "zero-cropland expansion (+0%)" is identical to the year 2000.

	NAWA	SSAfrica	CARussia	EAsia	SAsia	SEAsia	NAmerica	LAmerica	WEurope	EEurope	Oceania
**2000**											
Cereals	46	86	82	86	131	50	99	62	40	49	19
Roots	1	21	5	10	2	4	1	5	1	5	0
Sugarcrops	1	1	1	2	5	2	1	11	2	2	1
Pulses	4	18	1	4	24	3	4	10	2	1	2
Oilcrops	6	21	8	23	27	17	51	40	11	8	2
Vegetables and fruits	9	12	4	26	12	6	4	12	8	5	1
Other crops	4	17	4	7	19	10	8	15	2	1	1
**2050**											
**11%**											
Cereals	50	112	94	86	133	46	98	75	42	51	26
Roots	1	25	4	10	3	3	1	5	1	3	0
Sugarcrops	2	2	-	4	10	5	1	20	2	1	0
Pulses	5	16	1	4	28	3	6	9	1	0	3
Oilcrops	6	36	7	29	28	19	50	59	14	11	4
Vegetables and fruits	10	17	3	34	19	6	6	11	5	3	1
Other crops	5	21	2	11	31	10	13	11	3	0	1
**22%**											
Cereals	56	138	97	96	149	46	102	88	43	52	32
Roots	1	31	4	12	4	3	1	5	1	3	0
Sugarcrops	2	2	-	4	11	5	1	23	2	1	1
Pulses	5	20	1	4	31	3	6	10	1	0	4
Oilcrops	7	44	7	32	31	19	52	69	15	11	4
Vegetables and fruits	11	21	3	38	21	6	6	13	5	3	1
Other crops	5	26	2	12	34	10	14	13	3	0	1
**40%**											
Cereals	50	172	126	104	133	61	121	126	56	63	36
Roots	1	38	5	12	3	4	2	8	1	4	0
Sugarcrops	2	3	0	4	10	7	1	34	2	1	1
Pulses	5	25	1	5	28	4	8	15	2	0	4
Oilcrops	6	55	9	35	28	25	61	98	19	13	5
Vegetables and fruits	10	26	3	41	19	7	7	18	7	4	1
Other crops	5	33	3	13	31	13	16	19	4	0	1
**70%**											
Cereals	50	232	159	121	133	76	144	177	69	75	45
Roots	1	52	6	15	3	5	2	11	1	5	1
Sugarcrops	2	4	-	5	10	9	2	47	3	2	1
Pulses	5	34	2	5	28	5	9	21	2	0	5
Oilcrops	6	74	12	41	28	31	73	138	24	16	6
Vegetables and fruits	10	35	4	48	19	9	9	26	8	5	1
Other crops	5	44	4	15	31	16	20	26	5	1	1

Supplementary Table 8. Definition of world regions used in this study.

**NAWA**	Northern Africa and Western Asia	Algeria, Armenia, Azerbaijan, Cyprus, Egypt, Georgia, Iraq, Israel, Jordan, Kuwait, Lebanon, Libyan Arab Jamah., Morocco, Oman, Qatar, Saudi Arabia, Syrian Arab Republic, Tunisia, Turkey, United Arab Emirates, Western Sahara, Yemen
**SSAfrica**	Sub-Saharan Africa	Angola, Benin, Botswana, Burkina Faso, Burundi, Cameroon, Central African Rep., Chad, Dem. Rep. of Congo, Congo, Côte d'Ivoire, Djibouti, Equatorial Guinea, Eritrea, Ethiopia, Gabon, Gambia, Ghana, Guinea, Guinea-Bissau, Kenya, Lesotho, Liberia, Madagascar, Malawi, Mali, Mauritania, Mozambique, Namibia, Niger, Nigeria, Rwanda, Senegal, Sierra Leone, Somalia, South Africa, Sudan, Swaziland, United Rep. Tanzania, Togo, Uganda, Zambia, Zimbabwe
**CARussia**	Central Asia and Russian Federation	Kazakhstan, Kyrgyzstan, Russian Federation, Tajikistan, Turkmenistan, Uzbekistan
**EAsia**	Eastern Asia	China, Japan, Korea, Dem. Ppl's. Rep., Korea, Republic of, Mongolia
**SAsia**	Southern Asia	Afghanistan, Bangladesh, Bhutan, India, Iran(Islamic Rep. of), Nepal, Pakistan, Sri Lanka
**SEAsia**	South-Eastern Asia	Papua New Guinea, Brunei Darussalam, Cambodia, Indonesia, Lao People's Dem. Rep., Malaysia, Myanmar, Philippines, Thailand, East Timor, Viet Nam
**NAmerica**	Northern America	Canada, United States
**LAmerica**	Latin America & the Caribbean	Argentina, Belize, Bolivia, Brazil, Chile, Colombia, Costa Rica, Cuba, Dominican Republic, Ecuador, El Salvador, French Guiana, Guatemala, Guyana, Haiti, Honduras, Jamaica, Mexico, Nicaragua, Panama, Paraguay, Peru, Puerto Rico, Suriname, Trinidad and Tobago, Uruguay, Venezuela
**WEurope**	Western Europe	Austria, Belgium-Luxembourg, Denmark, Finland, France, Germany, Greece, Iceland, Ireland, Italy, Netherlands, Norway, Portugal, Spain, Sweden, Switzerland, United Kingdom
**EEurope**	Eastern & South-Eastern Europe	Albania, Belarus, Bosnia and Herzegovina, Bulgaria, Croatia, Czech Republic, Estonia, Hungary, Latvia, Lithuania, T.F. Yug. Rep. Macedonia, Republic of Moldova, Poland, Romania, Yugoslavia, Slovakia, Slovenia, Ukraine
**Oceania**	Oceania and Australia	Australia, New Zealand

Supplementary Table 9. Minima, maxima as well as the inner Quartiles (Q1 and Q3) and median of net-trade flows of all scenarios within a scenario group. Values are reported in Mtdm yr^{-1}. The Table indicates that, while the medians show a strong import dependency in a YIELDGAP with MEAT diets, cropland expansion can mitigate this dependency, stressing a potential trade-off between increased self-sufficiency and the sustainability challenges associated with the encroachment of farming into natural and semi-natural ecosystems, e.g. savannas.

		Crop products					Roughage equivalents				
Diet	Yield	Mtdm yr-1					Mtdm yr-1				
**VEGETARIAN**	**ORGANIC**	Min	Q1	Median	Q3	Max	Min	Q1	Median	Q3	Max
	North America & Oceania	-283	-161	-110	-33	-21	-92	-77	-31	-27	0
	Latin America	-263	-127	-87	-47	-32	-199	-175	-73	-63	0
	South-East Asia	-59	-40	-23	-14	-10	-24	-19	-8	-7	0
	Russia & Central Asia	-77	-33	-26	-10	-7	-13	-11	-5	-4	0
	Sub-Saharan Africa	-14	-4	133	250	354	-57	-42	-38	-21	0
	South Asia	-53	-33	-5	42	252	0	138	172	316	366
	Europe	-98	-43	-26	-15	-7	-31	-24	-11	-8	0
	North Africa & West Asia	95	110	126	134	172	-0	-0	0	48	72
	East Asia	-35	-17	-8	-2	67	-22	-19	-13	-8	0
**BAU**	**YIELDGAP**										
	North America & Oceania	-77	-34	-17	-6	68	-167	-59	-9	0	361
	Latin America	-227	-107	-55	-34	-6	-1.756	-459	-87	-17	0
	South-East Asia	-61	-41	-24	-11	-2	-203	-71	-14	-3	0
	Russia & Central Asia	-181	-79	-35	-17	-3	-118	-37	-6	-1	0
	Sub-Saharan Africa	-38	-9	-4	89	217	-903	-262	-43	-9	0
	South Asia	-58	-11	-2	63	146	0	37	118	299	451
	Europe	-75	-23	-8	1	58	-85	-55	-10	-2	0
	North Africa & West Asia	24	57	73	84	123	-0	0	55	138	182
	East Asia	-23	-14	-4	56	317	-4	-1	22	568	2.000
**BAU**	**FAO**										
	North America & Oceania	-208	-111	-72	-30	-3	-187	-106	-14	0	196
	Latin America	-277	-156	-102	-67	-31	-1.895	-504	-127	-18	0
	South-East Asia	-64	-48	-31	-20	-9	-185	-69	-18	-2	0
	Russia & Central Asia	-60	-29	-21	-11	-5	-98	-35	-7	-1	0
	Sub-Saharan Africa	-26	-5	85	163	293	-846	-239	-44	-8	0
	South Asia	-64	-6	-1	94	180	0	37	142	324	468
	Europe	-85	-41	-20	-14	38	-84	-56	-12	-2	0
	North Africa & West Asia	80	105	117	125	164	-0	0	70	154	201
	East Asia	-30	-18	-8	32	286	-4	-1	33	580	2.004
**MEAT**	**YIELDGAP**										
	North America & Oceania	-216	-157	-91	-59	-12	-450	-395	-103	-42	-4
	Latin America	-312	-168	-111	-74	-21	-982	-871	-242	-96	-9
	South-East Asia	-75	-46	-30	-21	-6	-23	-11	-7	73	247
	Russia & Central Asia	-145	-101	-60	-37	-8	-66	-60	-17	-7	-1
	Sub-Saharan Africa	-20	15	165	293	365	-105	-57	-44	-7	66
	South Asia	-9	20	176	253	440	22	208	452	1.136	1.216
	Europe	-94	-62	-43	-32	-8	-152	-125	-33	-12	-1
	North Africa & West Asia	57	85	127	140	205	-1	-0	55	153	168
	East Asia	-145	-101	-55	-35	-10	-29	-22	-16	43	96

Supplementary Methods

1. Modelling framework
2. Parameters and description of variants
    2.1. Household food demand
    2.2. Origin of livestock products in human diets
    2.3. Livestock feedstuff composition
    2.4. Grazing intensity
    2.5. Yields
    2.6. Cropland expansion

1. Modelling framework

BioBaM is a biophysical modelling framework that assesses the feasibility of scenarios, based on a unique combination of variants of the following parameters: a) human diets, b) origin of livestock products, c) composition of livestock feedstuff composition, d) yields, e) cropland expansion. All these variants are based on published forecasts or, in the case of human diets, recommendation of the USDA and HHS [4]. A scenario is each combination of these variants. All 500 scenarios are tested if supply covers demand and is, in negative cases, classified as cropland limited, grazing land limited or cropland and grazing land limited. Cases where supply of biomass is within 95% to 105% of demand are labelled as 'probably feasible', and as 'feasible' if it is larger than 105%. The model is applied to 11 world regions, and regional deficits in crops and roughage is assumed to be compensated by interregional trade. A schematic representation of the modelling framework is shown in Supplementary Figure 1.

2. Parameters and description of variants

2.1. Household food demand

We model per-capita food demand as food demand by households, i.e. including food wastes emerging during consumption at the household level. Supplementary Table 1 shows household food demand for the year 2000, which was used as baseline for the definition of the different variants. In 2050, we consider one business as usual variant (BAU) with household food demand differing between regions and four variants in which household food demand converges between regions (RICH, MEAT, VEGETARIAN and VEGAN). All variants for household food demand in 2050 are shown in Supplementary Table 2.

In the variant business as usual (BAU), per capita household food demand in 2050 is derived from FAO projections [3] and adjusted/completed according to projections given by IAASTD [6] in cases where FAO data was lacking or incomplete. Projections for fish consumption were taken from Wijkstrom [7], however we did not consider potential crop demand by aquaculture. In the variant RICH, per capita food demand of all 11 world regions converge until 2050 to per capita household food demand of North America in 2000 according to FAOSTAT (2014). Currently, North America has the highest overall food demand and the highest demand for livestock products of all regions (Supplementary Table 1).

The variants MEAT, VEGETARIAN and VEGAN are based on recommended diets according to USDA and HHS [8], and illustrate a diet including meat (MEAT), an ovo-lacto vegetarian diet (VEGETARIAN), and a vegan diet excluding all livestock products (VEGAN). MEAT, VEGETARIAN, and VEGAN start from an average per capita intake of 2,205 kcal cap $^{-1}$day^{-1}, based on the recommended total calorific intake for different age cohorts according to USDA and HHS [8], in combination with population age cohorts in 2050 according to UN [9]. In order to ensure a sufficient food supply for all persons and in all years, taking inequality in food access and yearly variations in food supply account, we add 10% to the resulting food intake, resulting in an average per capita intake of 2,425 kcal cap^{-1} day^{-1}. Based on this calorific

intake, we derive intakes for food product groups from USDA and HHS [8] and regroup food categories according to those applied in this study. Household food demand is calculated by adding an identical share of household food waste for all regions, amounting to global average household food waste in 2000, based on values given by Gustavsson et al. [4]. While this assumes that the share of household food waste remains equal on a global level, it implies future reductions of food waste in developed countries but an increase of food waste in developing countries, consistent with a variant of converging diets.

2.2. Origin of livestock products in human diets

For each household food demand variant (see above), we additionally modulate the share of livestock products derived from monogastric and ruminant livestock, respectively (Supplementary Table 4).

In the variant BAU, the share of ruminant and monogastric products in each region is in accordance with the FAO main projections for 2050 [3]. In the variant MONOGASTRIC, ruminant livestock products (meat and milk) are substituted completely by pigs, poultry and eggs, while the variant RUMINANT assumes that all livestock products from monogastrics (pigs, poultry and eggs) are substituted by ruminant meat and milk. As the variant VEGETARIAN does not allow a substitution of milk by meat from monogastrics in the variant MONOGASTRIC, milk is substituted by eggs in this case. All substitutions refer to food demand in terms of kcal cap^{-1} day^{-1}.

2.3. Livestock feedstuff composition

In terms of input-output ratios between feed intake and output of livestock products, we differentiate two variants: ROUGH, and GRAIN. For each of the livestock product categories covered in our study, a variant defines the quantity of a) feed from cropland (e.g. concentrates) and b) roughage feed required to produce one unit of livestock product, measured in tons of feed per ton of output (both in dry matter).

The projections for feed intake and product output for different world regions and livestock systems in 2050 are based on Bouwman et al. [10]. The more disaggregated world regions in Bouwman et al. (2005) are aggregated into the 11 world regions used in this study. Additionally, we aggregate the items grass, residues and fodder, and scavenging into the roughage feed category and project the values to 2050, assuming a continuation of the linear development from 1995 to 2030 according to Bouwman et al. [10]. We then modulate livestock feed composition in the variants ROUGH and GRAIN as follows.

In the variant GRAIN, we assume an acceleration of the trend to an increased share of concentrates in livestock feedstuff composition, operationalized as an increase of crop feed demand by 30% in all regions and an accordingly lower roughage demand, assuming a substitution weight of 0.5, i.e. 2 units of roughage are replaced by 1 unit of crops (measured in dry matter). To avoid extreme and rather unrealistic assumptions, we additionally set upper and lower boundary for crop and roughage intake [see 11 for details]. The variant ROUGH is a counter-trend variant, assuming that crops in ruminant diets are reduced by 50% in all regions compared to the trend scenario based on Bouwman et al. [10] and accordingly replaced by roughage, i.e. grazing (substitution weight as above). As Bouwman et al. (2005) assume increasing feed conversion ratios, the feed demand per kg of output in 2050 is lower than in the year 2000.

2.4. Grazing intensity

Due to the zero deforestation assumption, cropland is allowed to expand only into grazing land characterized by a productivity of >200gC $m2^{-1}$ yr^{-1} (Supplementary Figure 2). As a consequence, grazing land is assumed to shrink accordingly. In order to cover an eventual demand for roughage from grazing land by ruminants, the grazing intensity on the remaining grazing land is allowed to increase

up to a global average grazing intensity of 33% (defined as total grazed or harvested roughage per total aboveground productivity, Supplementary Table 7).

2.5. Yields

Variants for crop yields per area for each region are shown in Supplementary Table 5. The total range of crop yield variants is derived from major scenarios and considerations on how crop yields could develop until 2050 under the condition of specific land use practices (e.g. conventional or organic agriculture) and depending on technological optimism or pessimism. Future crop yields are delineated by the variant high yield as upper and the variant organic yields as lower boundary (Supplementary Table 3).

The variant FAO yield is derived from the latest FAO forecast on world agriculture towards 2030/2050 [3] and thus can be considered to represent the business as usual variant. The variant HIGH is in line with the scenario “Global Orchestration” of the Millenium Ecosystem Assessment[12]. Within the four scenarios of the MEA, the scenario “Global Orchestration” delineates the upper boundary of future increases in crop yields. The variant yield gap closed (YIELDGAP) is based on the yield gap analysis by Mueller et al. [13] and assumes that in all regions, crop yields will increase to the level attainable by industrial crop yield technologies already available (i.e. the optimal use of high yielding varieties, pesticides and synthetic fertilizers). The variant ORGANIC describes crop yields which are considered to be achievable in 2050 by organic agriculture (see explanation below).

In global average, there are only small differences between the variants FAO and YIELDGAP, which is reflected by the quite similar global biophysical option space shown in Figure 2 of main text. However, there are pronounced differences in terms of crop yields in 2050 between regions (Supplementary Table 5): While especially in Africa, Central Asia and Russia, and Eastern Europe, the variant YIELDGAP assumes considerably higher crop yields than the variant FAO, lower future increases in crop yields are assumed especially for Western Europe and North America, where yield gaps are already closed to a large degree according to the analysis by Mueller et al. [13].

Crop yields of the variant ORGANIC are derived as follows: In a first step, we assume that crop yields per harvest event (e.g. cereals or oil crops) in organic agriculture are 20% lower compared to conventional agriculture, as supported by two recent meta-studies on crop yields in organic agriculture [14,15]. In addition, it is assumed that 25% of the crop rotation in organic agriculture is required for legumes such as alfalfa [16] in order to replenish soil nitrogen, which we consider as an additional crop yield deduction. Considering the whole crop rotation including legumes, which cannot be used as food crops, this results in an assumed yield reduction by 40% of organic compared to industrial agriculture. However, it is important to consider that such a comparison refers only to organic compared to highly intensified agriculture with modern inputs such as high-yielding seed varieties, synthetic fertilizers and pesticides. It has been shown that in regions with high shares of traditional agriculture, crop yields can be increased without the use of industrial technologies (Pretty et al., 2011). Therefore, the yield deduction of 40% is only accounted for the share of highly intensive industrial agriculture in each world region, resulting in yield deductions of between 4% for Sub-Saharan Africa and 40% in North America compared to the variant FAO (Supplementary Table 5).

2.6. Cropland expansion

We consider five variants for the expansion of croplands, +0%, +11%, +22%, +40% and +70%, with the percentage denoting the global expansion of cropland until 2050 compared to global cropland areas in 2000. Region specific cropland areas for all variants are shown in Supplementary Table 6.

The variant +0% shows that croplands of the year 2000 still can support some scenarios in 2050. The variant +11% is in accordance with the FAO projections from Alexandratos and Bruinsma (2012) and

assumes a global expansion of croplands by 11%. Global studies of land suitable for cropland (IIASA and FAO, 2000; Ramankutty et al., 2002) suggest that cropland potentials are considerably larger than those assumed by the FAO. In the variant +22%, we thus double the global cropland expansion until 2050 assumed by the FAO.

The variants +40% and +70% are more extreme and assume that half (+40%) or all (+70%) grazing land of the highest productivity (grazing class I in Erb et al., 2007) will be converted into cropland until 2050. Additional land that would be necessary for infrastructure is considered in all variants, based on a extrapolation of current trends [17]. In the NAWA region, variants +40% and +70% were not possible and set equal to variant +11% (Supplementary Table 6). The zero-cropland expansion variant (0%) is identical to the year 2000.

Supplementary References

1. Bartholomé, E. & Belward, A. S. GLC2000: a new approach to global land cover mapping from Earth observation data. *Int. J. Remote Sens.* **26,** 1959–1977 (2005).
2. Erb, K.-H. *et al.* A comprehensive global 5 min resolution land-use data set for the year 2000 consistent with national census data. *J. Land Use Sci.* **2,** 191–224 (2007).
3. Alexandratos, N. & Bruinsma, J. *World Agriculture Towards 2030/2050. The 2012 Revision*. (Food and Agricultural Organization of the United Nations (FAO), 2012).
4. Gustavsson, J., Cederberg, C., Sonesson, U., Van Otterdijk, R. & Meybeck, A. *Global Food Losses and Food Waste. Extent, Causes and Prevention*. (Food and Agriculture Organization of the United Nations (FAO), 2011).
5. Haberl, H. *et al.* Quantifying and mapping the human appropriation of net primary production in earth's terrestrial ecosystems. *Proc. Natl. Acad. Sci.* **104,** 12942–12947 (2007).
6. IAASTD. *Agriculture at a Crossroads. International Assessment of Agricultural Knowledge, Science and Technology for Development (IAASTD), Global Report*. (Island Press, 2009).
7. Wijkstrom, U. N. Short and Long-term Prospects for Consumption of Fish. *Vet. Res. Commun.* **27,** 461–468 (2003).
8. USDA & HHS. *Dietary Guidelines for Americans, 2010*. (United States Department of Agriculture (USDA) and US Health and Human Services (HHS), 2010).
9. UN. *World Population Prospects: The 2012 Revision*. (Population Division of the Department of Economic and Social Affairs of the United Nations Secretariat, 2012).
10. Bouwman, A., Van der Hoek, K., Eickhout, B. & Soenario, I. Exploring changes in world ruminant production systems. *Agric. Syst.* **84,** 121–153 (2005).
11. Erb, K.-H., Mayer, A., Kastner, T., Sallet, K.-E. & Haberl, H. *The impact of Industrial Grain fed Livestock Production on Food Security: an extended literature review*. 132 (IFF Social Ecology, 2012).
12. *Ecosystems and Human Well-Being: Scenarios, Volume 2*. (Island Press, 2005).
13. Mueller, N. D. *et al.* Closing yield gaps through nutrient and water management. *Nature* **490,** 254–257 (2012).
14. de Ponti, T., Rijk, B. & van Ittersum, M. K. The crop yield gap between organic and conventional agriculture. *Agric. Syst.* **108,** 1–9 (2012).
15. Seufert, V., Ramankutty, N. & Foley, J. A. Comparing the yields of organic and conventional agriculture. *Nature* **485,** 229–232 (2012).
16. von Fragstein und Niemsdorff, P. & Kristiansen, P. in *Organic Agriculture. A Global Perspective* (eds. Kristiansen, P., Taji, A. & Reganold, J.) 53–82 (CABI, 2006).
17. Haberl, H. *et al.* Global bioenergy potentials from agricultural land in 2050: Sensitivity to climate change, diets and yields. *Biomass Bioenergy* **35,** 4753–4769 (2011).
